# Utilizing Supercritical CO_2_ for Bee Brood Oil Extraction and Analysis of Its Chemical Properties

**DOI:** 10.3390/foods13162486

**Published:** 2024-08-08

**Authors:** Pairote Wiriyacharee, Yongyut Chalermchat, Thanyaporn Siriwoharn, Wachira Jirarattanarangsri, Pipat Tangjaidee, Supakit Chaipoot, Rewat Phongphisutthinant, Hataichanok Pandith, Rattana Muangrat

**Affiliations:** 1 Division of Product Development Technology, Faculty of Agro-Industry, Chiang Mai University, Mae-Here, Muang, Chiang Mai 50100, Thailand; pairote.w@cmu.ac.th; 2 Multidisciplinary Research Institute, Chiang Mai University, Chiang Mai 50200, Thailand; supakit.ch@cmu.ac.th (S.C.); rewat.p@cmu.ac.th (R.P.); 3 Research Center of Microbial Diversity and Sustainable Utilization, Faculty of Science, Chiang Mai University, Chiang Mai 50200, Thailand; 4 Division of Food Process Engineering, Faculty of Agro-Industry, Chiang Mai University, Mae-Here, Muang, Chiang Mai 50100, Thailand; yongyut.c@cmu.ac.th; 5 Bioactive Compound Extraction Research Unit, Faculty of Agro-Industry, Chiang Mai University, Chiang Mai 50100, Thailand; thanyaporn.s@cmu.ac.th (T.S.); wachira.j@cmu.ac.th (W.J.); pipat.t@cmu.ac.th (P.T.); 6 Division of Food Science and Technology, Faculty of Agro-Industry, Chiang Mai University, Mae-Here, Muang, Chiang Mai 50100, Thailand; 7 Department of Biology, Faculty of Science, Chiang Mai University, Chiang Mai 50200, Thailand; hataichanok064@gmail.com

**Keywords:** supercritical CO_2_ extraction, tray-dried bee brood, quercetin, total flavonoids, fatty acids

## Abstract

To obtain oil from bee brood, which was dried using a tray drying method, this study used the supercritical CO_2_ extraction method. Extraction occurred at temperatures between 40–60 °C and low pressures of 180–220 bar for 1.5 h, with a high pressure of 600 bar for 1 h. The study investigated both the yield and chemical properties of the extracted bee brood oils. Supercritical CO_2_ extraction of tray-dried bee brood at 600 bar pressure demonstrated higher oil extraction efficiency compared to lower pressures (180–220 bar). At temperatures of 40–60 °C, total phenolic compounds increased while total flavonoids decreased. The extracted oil exhibited antioxidant activity, primarily due to quercetin. Despite decreased acid, iodine, and saponification values, peroxide value slightly increased but remained below 12 meqO_2_/kg of oil. The make-up of the fatty acids changed. At 600 bar, palmitic and oleic acids were the most common, while myristic, linoleic, and docosadienoic acids decreased. At 600 bar, eicosadienoic acid was absent. The defatted bee brood retained significant essential and non-essential amino acids, indicating its potential for further development as a protein source.

## 1. Introduction

Bee brood refers to the eggs, larvae, and pupae that are discovered within the cells of honeycombs [1,2]. Bee brood demonstrates noteworthy nutritional characteristics, including a substantial moisture content (74.6%), protein content (16%), fat content (3.7%), carbohydrates (4.1%), fiber (0.7%), and ash (0.9%) [3,4]. Additionally, it contains significant mineral elements, including potassium (K), sodium (Na), iron (Fe), zinc (Zn), copper (Cu), and manganese (Mn) [2]. Moreover, bee brood is rich in vitamins, featuring vitamin A, vitamin B1, vitamin B2, vitamin B6, vitamin B12, and vitamin C [3,4]. This nutritional composition positions bee brood as a viable alternative source of nutrients, making it an attractive option for consumption. The presence of essential vitamins and minerals underscores its potential health benefits. Bee brood is available alongside honey in the market [2], highlighting its recognition as a valuable dietary component. For optimal utilization, subjecting the bee brood to various culinary processes such as boiling, frying, or drying is recommended. Dried bee broods can be incorporated into various food products, including bread and soups, enhancing nutritional value and culinary diversity [4]. This underscores the versatility of bee brood as a nutritional resource, contributing to the broader discourse on sustainable and diverse dietary choices. Bee brood is rich in essential fatty acids such as oleic acid, palmitic acid, and stearic acid, offering diverse benefits [2,5] such as brain and body nourishment, fat and cholesterol reduction, increased red blood cell production, strengthened immunity against illnesses, inhibition of cancer cell growth, relief from allergies, and potential anti-aging effects. However, the challenge lies in consumers’ reluctance to consume whole bee brood due to its appearance. To address this, extraction and purification processes involving the isolation of protein, wax, and fat become crucial. While the extraction of fat or oil from bee brood for use in the food and cosmetics industries is currently limited, the identification of essential fatty acids in bee brood suggests significant potential to produce edible oils, food ingredients, and cosmetics, opening new possibilities for its utilization in various industrial applications.

Insect oil extraction, also known as defatting, employs various methods to remove lipids [6,7], for example, mechanical pressing [8,9], Soxhlet extraction [10], microwave-assisted solvent extraction [11], and supercritical CO_2_ extraction [12,13]. Some of these methods involve the use of solvents, while others, such as mechanical pressing, do not require solvents for the extraction process. Supercritical CO_2_ extraction has recently emerged as a widely adopted alternative for extracting oil from insects. Its appeal lies in its environmentally friendly characteristics, as the recycling of CO_2_ in the extraction process can help mitigate the greenhouse effect [6]. Supercritical CO_2_ is preferred due to its unique solvation and solvent selectivity, which can be adjusted by manipulating temperature and pressure [14]. Generally recognized as safe (GRAS), CO_2_ possesses low viscosity, non-toxicity, cost-effectiveness, easy separation, and high solvent power. Additionally, CO_2_ can be easily removed from the extracted products, ensuring the absence of residual organic solvents [15]. This technique aids in maintaining the stability and quality of heat-sensitive natural components without compromising their bioactivity [15,16]. 

Another challenge of this study is to add value to bee broods and increase income for Thai beekeeping farmers by exploring oil extraction from bee broods during non-honey-producing periods in northern Thailand. This approach offers alternative income sources, ensuring a consistent revenue stream throughout the year. Extracting bee brood oil, which can be used in functional food ingredients, medicines, or cosmetics, is essential for providing Thai beekeeping farmers with viable year-round income options. The supercritical CO_2_ extraction method is crucial for preserving the quality and composition of insect oils [13,17]. This method effectively maintains the stability of essential biological substances, resulting in minimal alterations and improved oil quality. Recognized as a green technology by the food, dietary supplement, pharmaceutical, and cosmeceutical industries for its environmentally friendly characteristics, this study aimed to investigate the feasibility of using supercritical CO_2_ for extracting oil from bee broods by adjusting extraction parameters such as temperature and pressure. This study also examined the yield, chemical properties, and stability of the extracted oil. The chemical properties measured included acid value, iodine value, saponification value, peroxide value, identification and quantification of phenolic compounds, total flavonoid and carotenoid content, antioxidative activities (DPPH, ABTS, and FRAP assays), and fatty acid composition.

## 2. Materials and Methods

### 2.1. Sample Preparation

Bee brood samples, initially blanched and frozen, were acquired from farmers in Phrae Province, Thailand. The thawing process involved using a ratio of 3 kg of bee brood sample to 3 L of water. Following thawing, the bee brood samples were drained and dried with tissue paper. The prepared bee brood samples were spread evenly to a uniform thickness of approximately 0.7 cm on aluminum trays covered with foil. Two of these aluminum trays, each containing bee brood samples, were then placed on each stainless steel tray within the tray dryer. The drying process involved a total of 6 stainless steel trays per batch, each measuring 53 × 72 × 3 cm. The bee brood samples were dried with hot air in a tray dryer (86 × 94 × 200 cm, 0.4 kW/380 V, Kluaynamthai Trading Group Co., Ltd., Bangkok, Thailand) at 70 °C for 24 h. After each batch of the tray drying process, the weight of the tray-dried bee brood samples was one-third of the original weight of the bee brood samples before drying. The dried bee brood samples were ground to reduce their size using a blender (Sharp EM-ICE2 Model, Bangkok, Thailand). The average size of the ground samples was determined by passing them through a sieve shaker, which operated at an amplitude of 1.75 mm with 3 s intervals for 20 min, resulting in an approximate average size of 0.90 mm. Next, the ground samples were sealed in vacuum bags and subjected to analysis and extraction using the supercritical CO_2_ extraction method.

### 2.2. Chemicals and Reagents

Lanna Industrial Gases Co., Ltd. supplied carbon dioxide in cylinders (99.99% purity) (Chiang Mai, Thailand). Other chemicals and reagents of analytical grade were obtained commercially. RCI Labscan Ltd., Bangkok, Thailand, provided hexane, dichloromethane, acetonitrile, liquid Wijs solution, hydrochloric acid, sulfuric acid, and ethanol. Supelco 37 component FAME mix, boron trifluoride, 2-thiobarbituric acid, acetonitrile, standard gallic acid, potassium hydroxide, potassium iodide, sodium thiosulphate, soluble starch, ammonium thiocyanate, DPPH (2,2-diphenyl-1-picrylhydrazyl), and ABTS (2,2′-azinobis-3-ehtylbenzothiazoline-6-sulfonic acid) were obtained from Sigma-Aldrich Chemical Co., Ltd., Burlington, MA, USA, and Qrec, Auckland, New Zealand. Merck, Darmstadt, Germany, provided Folin–Ciocalteu reagent, quercetin, and HPLC-grade methanol.

### 2.3. Supercritical CO_2_ Extraction 

**Figure 1:**
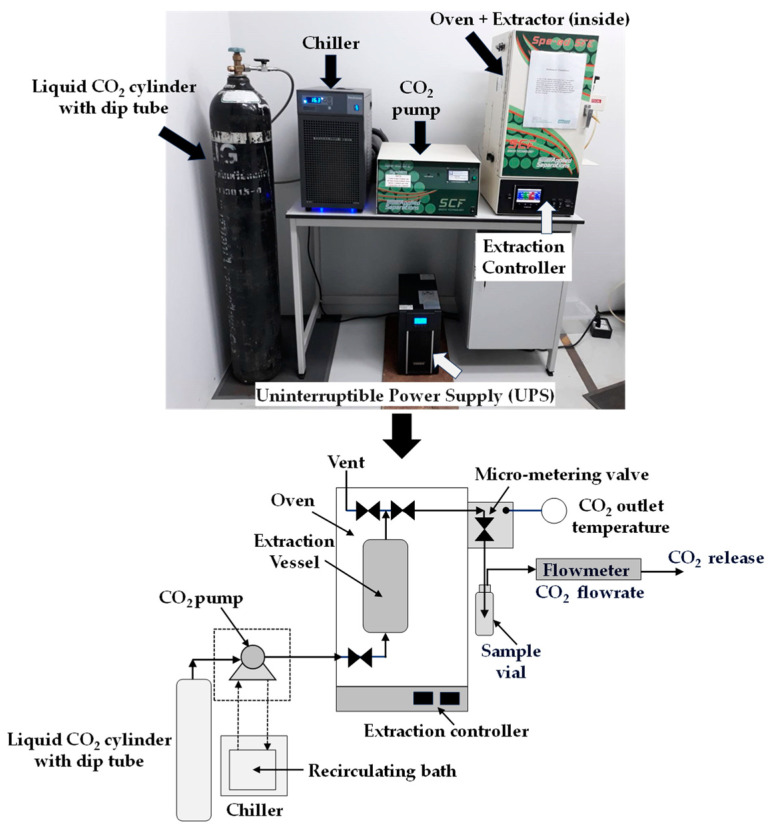
A schematic of the supercritical CO_2_ extraction process.

Supercritical CO_2_ extraction was performed on tray-dried bee brood samples following grinding. Prior to extraction, tray-dried bee brood samples of specific sizes were selected and processed using a Retsch AS200 vibratory sieve shaker (Retsch, Haan, Germany). The samples, weighing 25 g, were then placed inside a 50 mL extracting vessel of the Spe-edTM SFE-2 extractor system (Applied Separations, Allentown, PA, USA), ensuring their complete coverage. A schematic of the supercritical CO_2_ extraction process is shown in Figure 1. 

Extraction was carried out at 40, 50, and 60 °C under pressures of 180, 200, and 220 bar for 1.5 h. Additionally, extraction was performed at a higher pressure of 600 bar over the same temperature range for 1 h. The extracted oil was dissolved in supercritical CO_2_, separated by lowering the pressure, and collected in a separator. The sample collection bottle, fitted with a septum cap, was positioned within the pointed tube adjacent to the extraction vessel after the specified extraction time. The gradual opening of the valve at the bottom of the extraction vessel led to a decrease in pressure. Although the supercritical CO_2_ extractor did not feature a pressure measurement system, the pressure was expected to approximate atmospheric pressure at the outlet of the pointed pipe leading to the sample collection bottle. The Spe-ed™ SFE-2 extractor system includes a controller for both CO_2_ flow rate and outlet temperature. At the exit of the extraction vessel, there is an exit valve followed by a micro-metering valve to control the CO_2_ flow rate, which is measured by an electronic flowmeter at the exit of the sample collection flask. The extractor was set with a CO_2_ flow rate of 3 L/min and an outlet temperature of 110 °C to prevent the CO_2_ from becoming a liquid. A bent tube was inserted into the septum cap of the sample collection bottle to collect the extracted oil, while the CO_2_ was separated and released outside, as shown in Figure 1. Oil yield was calculated as a percentage using Equation (1).

(1) $$Oil\;yield=\frac{weight\;of\;the\;extracted\;oil\;\left(g\right)\times 100\%}{weight\;of\;the\;dried\;and\;ground\;bee\;brood\;sample\;(g)}$$

Each extracted oil sample was stored in an amber flask at 4 °C for further analysis.

### 2.4. Chemical Properties of Bee Brood and Extracted Oil

Bee brood samples underwent analysis for moisture, protein, lipid, ash, crude fiber, and carbohydrate content following the method in AOAC (2000) [18]. The acid, iodine, saponification, and peroxide values of the extracted oil samples were determined as shown below.

The acid value was determined following the AOCS official method Cd 3a-63 [19]. A 2 g oil sample was mixed with 20 mL of neutral ethyl alcohol, boiled for 5 min, then 1 mL of phenolphthalein was added. The mixture was then titrated with 0.1 N potassium hydroxide. The acid value was calculated as follows:

(2) $$\mathrm{Acid}\;\mathrm{value}\;(\mathrm{mg}\;\mathrm{KOH}/g)=\frac{56.1\times V\times N}{W}$$

where V is the titration volume of 0.1 N potassium hydroxide, N is the concentration of potassium hydroxide (0.1 N), and W is the oil sample weight (g).

The iodine value was determined using the AOCS official method Cd 1d-92 [19]. A 1 g oil sample was placed in a 250 mL Erlenmeyer flask. After adding 20 mL of cyclohexane-acetic acid and shaking, 25 mL of Wijs solution was added and the flask was kept in the dark for 1 h. Then, 20 mL of 15% potassium iodide and 150 mL of distilled water were added. The mixture was titrated with 0.1 N sodium thiosulfate until pale yellow, and then with 1 mL of starch indicator until the blue color disappeared. A blank was also prepared without the oil sample. The iodine value was calculated as follows:

(3) $$\mathrm{Iodine}\;\mathrm{value}\;(g\;I_{2}/100\;g)=\frac{12.6\times \left(V_{blank}-V_{sample}\right)\times N}{W}$$

where V_sample_ is the titration volume of sodium thiosulphate used in the extracted oil sample, V_blank_ is the titration volume of sodium thiosulphate used in the blank solution, N is the concentration of sodium thiosulphate (0.1 N), and W is the oil sample weight (g).

The saponification value was determined according to the AOCS method Cd 3-25 [20]. A 1 g sample of oil was weighed into a 250 mL flask, and 25 mL of 0.1 N alcoholic potassium hydroxide was added. The mixture was boiled under reflux until it became clear and was then titrated with 1.0 N HCl using phenolphthalein as an indicator. A blank test was performed without the oil sample. The saponification value was calculated as follows:

(4) $$\mathrm{Saponification}\;\mathrm{value}\;(\mathrm{mg}\;\mathrm{KOH}/g\;\mathrm{oil})=\frac{56.1\times \left(V_{blank}-V_{sample}\right)\times N}{W}$$

where V_sample_ is the titration volume of hydrochloric acid used in the extracted oil sample, V_blank_ is the titration volume of hydrochloric acid used in the blank solution, N is the concentration of hydrochloric acid (1.0 N), and W is the oil sample weight (g).

The peroxide value was determined using the AOCS official method Cd 8-53 [21]. A mixture was prepared by combining 2 g of oil with a chloroform–acetic acid solution (3:2 *v*/*v*), followed by the addition of 0.5 mL of saturated potassium iodide. The solution was shaken and left in the dark for 5 min. After this period, 30 mL of distilled water was added. The resulting mixture was titrated with 0.01 N sodium thiosulphate until a yellow endpoint was reached, with 1 mL of starch solution used as an indicator. A blank test was conducted without oil. The peroxide value was calculated as follows:

(5) $$\mathrm{Peroxide}\;\mathrm{value}\;({\mathrm{meqO}}_{2}/\mathrm{kg}\;\mathrm{oil})=\frac{\left(V_{sample}-V_{blank}\right)\times N\times 1000}{W}$$

where V_sample_ is the titration volume of sodium thiosulphate used in the extracted oil sample, V_blank_ is the titration volume of sodium thiosulphate used in the blank solution, N is the concentration of sodium thiosulphate (0.01 N), and W is the oil sample weight (g).

### 2.5. Fatty Acid Composition Analysis

The fatty acid compositions were determined by converting a sample of the extracted oil into the methyl esters of fatty acids, following the methods outlined by Kim and Oh [22] and Mattioli et al. [23]. To initiate the process, 0.5 g of the extracted oil was weighed and mixed with 5 mL of methanol containing 0.5 M sodium hydroxide. The resulting mixture was refluxed for 5 min at 110–120 °C and then allowed to settle at room temperature. Subsequently, a 20% concentration of boron-trifluoride in methanol was poured into the solution, followed by another 5 min reflux. After cooling to ambient temperature, the mixture was combined with 5 mL of hexane and 10 mL of saturated salt (NaCl), and the layers were separated through shaking. The upper solution was then filtered through a 0.45 µm pore size filter, diluted tenfold with hexane, and injected into a Gas Chromatography system (Nexis GC-2030, Shimadzu Co., Kyoto, Japan). The chromatography utilized an RT^®^-2560 column (biscyanopropyl polysiloxane) with the following dimensions: 100 mm in length, 0.25 mm in inner diameter, and 0.2 µm in film thickness (Restek^®^, Centre County, PA, USA), equipped with a flame ionization detector (FID). The injector and detector temperatures were set at 250 and 260 °C, respectively. Over a period of 45 min, the column temperature was programmed to increase from 140 °C (with a 4 min hold time) to 230 °C at a rate of 3 °C/min. Helium served as the carrier gas, maintaining a constant flow rate of 1.2 mL/min.

For the comparison of retention times, reference standards (37-component FAME mix) were used under identical conditions. The results were expressed in g of fatty acids per 100 g of oil.

Equations (6)–(10) depict computations for various fatty acid composition indices, which evaluate the dietary influence of foods on cardiovascular health. Specifically, Equation (6) calculates the polyunsaturated/saturated fatty acids (PUFAs/SFAs) ratio by dividing the total polyunsaturated fatty acids by the total saturated fatty acids [24,25].

(6) $$\mathrm{PUFAs}/\mathrm{SFAs}=\frac{\sum Polyunsaturated\;fatty\;acids}{\sum Saturated\;fatty\;acids}$$

Equation (7) presents the omega-6/omega-3 ratio (n-6/n-3), an index of nutritional quality advocated by Dal Bosco et al. [25] and Simopoulos [26]. This ratio is determined by summing up specific n-6 fatty acids and dividing them by the sum of corresponding n-3 fatty acids. A lower n-6/n-3 ratio is considered beneficial due to its potential to reduce the risk of chronic diseases globally, emphasizing the nutritional value of foods for diverse populations.

(7) $$\text{n-6}/\text{n-3}=\frac{\left(C18:2\text{n-6}+C20:2\text{n-6}+C20:3\text{n-6}+C20:4\text{n-6}\right)}{\left(C18:3\text{n-3}+C20:3\text{n-3}+C20:5\text{n-3}+C22:5\text{n-3}+C22:6\text{n-3}\right)}$$

Equation (8) presents the index of atherogenicity (IA), computed as the ratio of certain saturated fatty acids to total monounsaturated and polyunsaturated fatty acids. This metric offers valuable information on the atherogenic potential of dietary fatty acids, emphasizing the equilibrium between distinct saturated and unsaturated fatty acids. A higher IA suggests a potential adverse impact on cardiovascular health, as elucidated by Chen and Liu [24] and Dal Bosco et al. [25].

(8) $$\mathrm{IA}=\frac{\left(C12:0+(4\times C14:0)+C16:0\right)}{\sum (MUFAs+PUFAs)}$$

Following Equation (9), the index of thrombogenicity (IT) is elucidated, wherein it is computed as the proportion of particular saturated fatty acids in relation to a composite of monounsaturated, n-6, and n-3 fatty acids. This index serves as a tool for evaluating the thrombogenic propensity of dietary fatty acids, considering the equilibrium among saturated, monounsaturated, and polyunsaturated fatty acids, as discussed by Chen and Liu [24] and Dal Bosco et al. [25].

(9) $$\mathrm{IT}=\frac{\left(C14:0+(C16:0+C18:0\right)}{[\left(0.5\times MUFAs\right)+(0.5\times \sum \text{n-6})+(3\times \sum \text{n-3})+\frac{\sum \text{n-3}}{\sum \text{n-6}})]}$$

Equation (10) introduces the hypocholesterolemic/hypercholesterolemic (H/H) ratio, emphasizing the interplay between dietary fatty acids and plasma low-density lipoproteins. This ratio is determined by summing up hypocholesterolemic fatty acids (C18:1n-9 and PUFAs) and dividing this sum by total hypercholesterolemic fatty acids (C12:0, C14:0, and C16:0). The calculation methodology and insights into this ratio are elaborated by Chen and Liu [24] and Dal Bosco et al. [25].

(10) $$H/H\;\mathrm{ratio}=\frac{(C18:1+\sum PUFAs)}{\left(C12:0+C14:0+C16:0\right)}$$

### 2.6. Determination of Total Phenolic Compound Content

The determination of total phenolic compound content was conducted with modifications to the methodology proposed by Houshia et al. [27]. In this adapted procedure, 1 g of the extracted bee brood oil sample was dissolved in 1 mL of hexane and subsequently combined with 2 mL of 80% methanol. The mixture was vigorously shaken in a vortex for 15 min and then centrifuged at 5000 rpm for 10 min. The resulting methanol layer was carefully pipetted (20 μL) and mixed thoroughly with 100 μL of 10% Folin–Ciocalteu solution. Following a 5 min interval, 20 μL of 7.5% *w*/*v* sodium carbonate (Na_2_CO_3_) was added to the mixture and shaken well before allowing it to stand at room temperature for 1 h. After completing all preparations, 140 µL of the mixture solution was obtained. A small volume of this mixture was transferred to the 96-well plate using a micropipette for absorbance measurement at a wavelength of 725 nm, using an automated microplate reader (SpectraMax i3x, Molecular Devices, San Jose, CA, USA). The absorbance value was compared to a blank, which was prepared by mixing 20 µL of distilled water with 100 µL of Folin–Ciocalteu solution and 20 µL of 7.5% (*w*/*v*) sodium carbonate. The total phenolic content was measured using the standard curve of gallic acid concentrations (0–180 mg/L; y = 0.0049x + 0.0383; R^2^ = 0.996). The results, expressed as µg of gallic acid equivalents (GAE) per g of oil, indicate the total phenolic compounds present in the oil samples.

### 2.7. Identification and Quantification of Phenolic Compounds Using HPLC

The identification and quantification of phenolic compounds in both the dried bee brood and the extracted oil samples were carried out utilizing a High-Performance Liquid Chromatography (HPLC) 1200 series diode-array detector apparatus (Agilent Technologies, Santa Clara, CA, USA). To prepare extract samples from the dried bee brood and extracted oil, a concentration of 1000 mg/mL was achieved by mixing the samples with methanol (HPLC grade) and subjecting them to sonication at high-frequency waves for 1 h. After extraction, the samples were filtered through a 0.22 μm nylon membrane filter and packaged in amber HPLC vials with a volume of 2 mL. To analyze the quercetin content, 10 μL sample solutions were taken. In this method, the composition of the mobile phase remains constant throughout the analysis. The mobile phases consisted of (A) 1.0% *v*/*v* phosphoric acid, (B) MeOH + 1.0% *v*/*v* phosphoric acid, and (C) acetonitrile + 1.0% *v*/*v* phosphoric acid in a ratio of 70:5:25. The separation was carried out on an Agilent Eclipse XDB C18 column (4.6 mm × 150 mm, 5 μm). The column temperature was set at 25 °C. The flow rate was set at 1.0 mL/min for a duration of 15 min, with detection at a wavelength of 254 nm. Establishing a standard curve for quercetin involved preparing solutions with concentrations ranging from 15.625 to 500 μg/mL, dissolved in methanol. These solutions, injected into the HPLC apparatus at a volume of 10 µL, demonstrated linearity (y = 63.2102 + 472.3574, R^2^ = 0.997), as shown by the standard curve of quercetin and the chromatogram of quercetin, illustrated in Figure S1 and Figure S2, respectively.

### 2.8. Determination of Total Flavonoid Content

The total flavonoid content in the extracted bee brood oil samples was determined using a modified aluminum chloride colorimetric method based on Chang et al. [28]. To achieve this, 0.5 g of the extracted bee brood samples was combined with 500 µL hexane and subsequently mixed with 1 mL of 80% methanol. After vortexing the mixture for 15 s to obtain the supernatant, 250 µL of each sample supernatant was added to 25 µL of a 10% *w*/*v* aluminum chloride aqueous solution, 25 µL of a 1.0 M potassium acetate solution, and 700 µL of deionized water, respectively. The resulting mixture was then incubated in the dark at room temperature for 30 min. The presence of flavonoids was identified through spectrophotometric measurements using a spectrophotometer (Shimadzu: UV1800, Kyoto, Japan) set at a wavelength of 415 nm. Quercetin served as the standard compound, and the results are expressed in µg of quercetin equivalent per g of oil sample. The total flavonoid content of extracted bee brood oil was analyzed, with three replicates taken for each sample. 

### 2.9. Determination of Total Carotenoid Content

The total carotenoid content was determined according to the method of Chinarak et al. [29]. A 1.0 g oil sample was mixed with a 1:10 (*w*/*v*) solvent (petroleum ether: acetone: distilled water; 15:75:10; *v*/*v*/*v*) and homogenized for 2 min at 25 °C. After centrifugation at 3000× *g* for 30 min at 25 °C, the supernatant was filtered using Whatman No. 1 paper and adjusted with petroleum ether. Samples were analyzed at 470 nm using a spectrophotometer (Shimadzu: UV1800, Kyoto, Japan). Carotenoid content was calculated using a β-carotene extinction coefficient of 2400 in petroleum ether and expressed as μg β-carotene equivalents per g oil. The total carotenoid content was calculated according to the following equation.

(11) $$\mathrm{Carotenoids}\;\mathrm{content}\;(\mu g/g)=\frac{Abs\times V\times 10^{4}}{A_{1cm}\times W(g)}$$

where Abs is the absorbance value, V is the total amount of the extract (mL), A_1cm_ is 2400 (β-carotene extinction coefficient in petroleum ether), and W is the sample weight (g).

### 2.10. Amino Acid Analysis

The analysis of amino acids employing the ARACUS Amino Acid Analyzer (Membrapure, Hennigsdorf, Germany) underwent hydrolysis with hydrochloric acid, as described by Vanderplanck et al. [30]. Initially, 100 mg of dried bee brood samples were placed in a PTFE container. Subsequently, 10 mL of 6M HCl and 1–2 drops of phenol were added, followed by a 15 min nitrogen gas purge to eliminate oxygen. The sample was then hydrolyzed at 110 °C for 24 h, cooled, filtered into a 50 mL volumetric flask, rinsed with deionized water, and brought up to 50 mL. The resulting clear hydrolysate (2 mL) was aliquoted and evaporated to dryness under a rotary vacuum. Following this, 2 mL of the sample dilution buffer (SDB) was introduced to dissolve the dried sample. The sample was then filtered through a 0.45 µm syringe filter into a vial for analysis, and a clean extract was injected (20 µL) into the amino acid analyzer. Detection was performed using a photometer at 570 nm for primary amino acids and 440 nm for secondary amino acids, specifically proline. iControl and AminoPeaks software were employed for instrument control and data analysis.

### 2.11. Determination of Free Radical Scavenging (ABTS) Assay

The ABTS•+ test for antioxidant activity was analyzed following the methods of Re et al. [31], where ABTS was dissolved in phosphate-buffered saline at 0.01 mol/L with pH 7.4 to obtain the last concentration at 7 mmol/L. The ABTS•+ solution was made when the prepared ABTS solution was reacted with potassium persulfate at the concentration of 2.45 mmol/L in the ratio of 1:1 and left in darkness at room temperature for 16 h before use. After that, ABTS•+ solution was diluted with 80% methanol to make it reach an absorption of 0.7 ± 0.02 at a wavelength of 734 nm. Approximately 0.1 g of extracted oil sample was mixed with 2 mL of methanol for 15 s using a vortex shaker. The sample mixture was centrifuged at 5000 rpm for 10 min. Then, 20 μL of supernatant was mixed with 200 μL of diluted ABTS•+ solution before leaving it to react for 5 min. Subsequently, it was measured for absorption at the wavelength of 734 nm by a microplate reader (Spark, Tecan, Männedorf, Switzerland). Methanol solutions of known Trolox concentrations from 0 to 240 μmol/L were used for the calibration curve (y = −0.0024x + 0.6755; R^2^ = 0.9913). The antioxidant capacity was calculated from the calibration curve using the Trolox standard and expressed as µmol Trolox equivalents (TEAC) per g of oil.

### 2.12. Determination of Free Radical Scavenging (DPPH) Assay

The DPPH• test of antioxidant activity was analyzed following the methods of Brand-Williams et al. [32]. About 0.1 g of the extracted oil sample was mixed with 2 mL of methanol for 15 s using a vortex shaker. The sample mixture was centrifuged at 5000 rpm for 10 min. Next, 20 μL of supernatant was mixed with 200 μL of DPPH solution in methanol. The mixture was stored for 10 min in a dark place. Absorbance at 515 nm was measured using a microplate reader (Spark, Tecan, Männedorf, Switzerland). Methanol solutions of known Trolox concentrations from 0 to 480 μmol/L were used for the calibration curve (y = −0.0012x + 0.6921; R^2^ = 0.9961). The antioxidant capacity was calculated from the calibration curve using the Trolox standard and expressed as µmol Trolox equivalents (TEAC) per g of oil.

### 2.13. Determination of Ferric Reducing Antioxidant Power (FRAP)

The FRAP of the extracted oil was determined [33,34]. The FRAP reagent (Fe (III) solution and TPTZ) was prepared by the mixture of 0.1 mol/L acetate buffer (25 mL), a solution of 10 mmol/L TPTZ (2,4,6-tri(2-pyridyl)-s-triazine) (2.5 mL), and a solution of 20 mmol/L ferric chloride (2.5 mL). About 0.1 g of the extracted oil sample was mixed with 2 mL of methanol for 15 s using a vortex shaker. The sample mixture was centrifuged at 5000 rpm for 10 min. Next, 20 μL of supernatant was mixed with 200 μL of FRAP reagent and incubated in the water bath for 30 min at 37 °C. Then, the absorbance of the colored sample solution was measured at 593 nm using a microplate reader (Spark, Tecan, Männedorf, Switzerland). A standard curve was prepared using different concentrations of Trolox. The Trolox solution (ranging from 0 to 480 μmol/L) was employed as a standard for the calibration curve (y = 0.0031x + 0.039; R^2^ = 0.9971), and the results were stated in µmol Trolox equivalents (TEAC) per g of oil. Analyses were performed in triplicate on each sample.

### 2.14. Determination of Stability 

The extracted oil samples were subjected to oxidative stability analysis utilizing a modified method based on Przybylski et al. [35]. These oil samples were carefully kept in dark brown bottles and stored at two different temperatures: 25 °C and an accelerated storage temperature of 55 °C. The storage period was 60 days, during which their peroxide values were assessed to measure oxidative stability. 

### 2.15. Statistical Analysis 

The data were analyzed using statistical methods and analysis of variance (ANOVA). The difference in the average was compared using Duncan’s New Multiple Range Test at a statistical confidence of 95%. In addition, to compare the mean values of properties between two samples, *t*-test analysis was used.

## 3. Results

### 3.1. Proximate Composition of Bee Brood

Bee brood samples were tray-dried at 70 °C for 24 h, resulting in an amount of dried bee brood samples obtained of 23.92%. Following the drying process, the dried bee brood underwent proximate analysis to determine moisture, protein, lipid, ash, crude fiber, and carbohydrate content, as detailed in Table 1. The obtained dried bee brood samples were then used for oil extraction using supercritical CO_2_.

After completing the tray drying process for bee brood, the resulting dried product displayed a lipid content of 34.64%. This underscores the advantages of employing drying pretreatment prior to oil and protein extraction. Nonetheless, it is crucial to exercise caution in regulating both drying temperature and time to maintain the quality of lipids. Subsequently, supercritical CO_2_ was used in the extraction process to obtain oil from the dried bee brood. The following section highlights the chemical properties of the extracted oil.

### 3.2. Oil Extraction Using Supercritical CO_2_

The impact of extraction temperature, pressure, and various extraction setups on crude oil yield from tray-dried bee brood was significant. Table 2 shows the percentage yield of crude oil obtained through supercritical CO_2_ extraction from tray-dried bee brood. The results demonstrated that increasing extraction temperature correlated with a decrease in crude oil yield, while increasing extraction pressure led to higher yields. At higher temperatures, CO_2_ becomes less effective at dissolving oil, resulting in lower yields. Conversely, higher extraction pressure enhances CO_2_’s ability to dissolve oil and facilitates higher mass transfer rates and deeper penetration into the material, ultimately increasing oil extraction. Similarly, Kim et al. [12] found that high extraction pressures promoted more efficient oil extraction from insect meals, such as black soldier flies, using supercritical CO_2_. Purschke et al. [13] also reported that higher pressure enhanced the solubility of mealworm oil in supercritical CO_2_, enabling faster extraction kinetics.

Table 3 illustrates the proximate composition of defatted tray-dried bee brood obtained through supercritical CO_2_ oil extraction. The extracted oil from tray-dried bee brood through supercritical CO_2_ extraction at a low-pressure range of 180–220 bar resulted in a limited oil yield, causing residual oil to persist in the defatted bee brood meal. Nonetheless, the defatted bee brood contained a substantial protein content, ranging from 42.33% to 45.29%. Defatted bee brood meal emerges as a promising protein source for integration into the food industry. These results align with Cruz et al. [36], who noted that supercritical CO_2_ extraction is one of the most effective methods for obtaining oil and generating crude protein, the key component of insect meals. Research focused on producing defatted meals free from organic solvent residues highlights the importance of this technique. Defatting the meal is crucial for ensuring its suitability as an ingredient in food and feed products enriched with insect-based proteins. 

### 3.3. Acid, Iodine, Saponification, and Peroxide Values of Extracted Oil from Dried Bee Brood

The results revealed that the extraction temperature and pressure notably impacted the acid, iodine, and saponification values of the oil extracted from the tray-dried bee brood (Table 2). This is because the temperature has a dual influence on the extraction process, depending on whether the solute vapor pressure or solvent density is dominant. As extraction temperature increases under low pressures, the increased kinetic energy of the solute molecules can lead to a decrease in their solubility in CO_2_ [37], resulting in lower solubility of free fatty acids and a lower acid value in the extracted oil samples. The acid value serves as a crucial quality indicator for fats or oils, reflecting the presence of free fatty acids and other non-lipid acidic compounds, such as acetic acid. Various factors contribute to crude oil’s acidity, such as the extraction process employed and the freshness of the raw materials [38]. A reduced acid value typically signifies the purity and appropriateness of oils or fats, whereas an elevated value is linked to rancidity. Therefore, if extracted bee brood oil is to be developed for consumption in future research, its acid value may need to be reduced. The acid value was consistent with the FAO/WHO standard of 4 mg KOH/g [39]. Similarly, Sasongko et al. [40] reported that the acid value of edible oil should not exceed 4 mg KOH/g. 

The iodine value is indeed a measure of the amount of unsaturated fatty acids in the oil. Increased extraction temperature and pressure led to a decrease in the iodine value, indicating a reduction in unsaturated fatty acids. Consequently, this suggested that higher extraction temperatures and pressures may alter the fatty acid composition of the extracted oil, potentially leading to a higher proportion of saturated fatty acids [41]. 

Moreover, saponification of supercritical CO_2_-extracted oil involves breaking down the triglycerides in the oil into glycerol and fatty acids. The saponification value serves to measure the amount of fatty acids in oil. An increased saponification value indicates the presence of fatty acids with lower molecular weights [42]. In this study, the saponification value decreased as the extraction temperature increased, primarily due to a reduced proportion of triacylglycerols with lower molecular weights dissolved in the extracted oils [43,44].

The peroxide value assesses the degree of rancidity in oil throughout processing, extraction, and storage. Additionally, it serves as a tool for monitoring the quality and stability of fats and oils. A lower peroxide value indicates higher oil quality [42]. In the present study, the peroxide value decreased with increased extraction pressure from 200 to 220 bar under a constant extraction temperature. Although increasing the extraction temperature at a constant pressure resulted in an increase in the average peroxide value, these values were not significantly different (*p* < 0.05), as detailed in Table 2.

The comprehensive experimental results demonstrated a noteworthy influence of higher extraction temperatures at each extraction pressure level on decreasing the acid, iodine, and saponification values in the oil extracted from the tray-dried bee brood.

### 3.4. Stability of Extracted Oil

**Figure 2:**
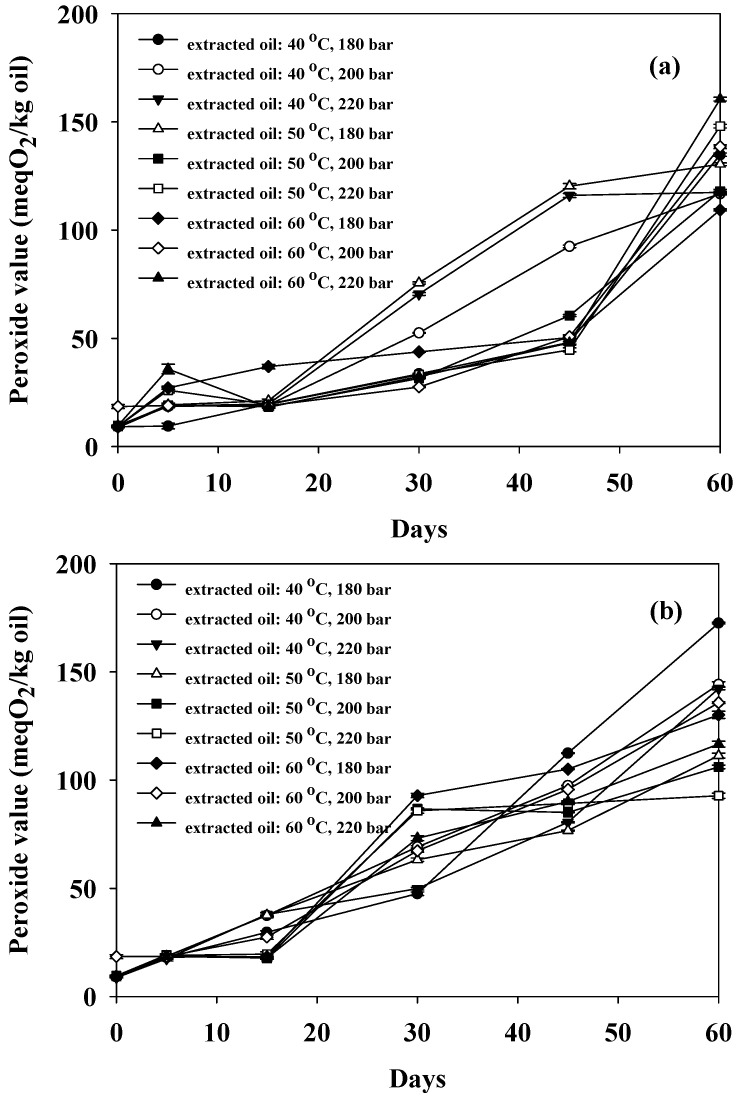
Peroxide values of tray-dried bee brood oil extracted with supercritical CO_2_ and stored at (**a**) 25 and (**b**) 55 °C for 60 days. Samples were extracted at different temperatures (40, 50, and 60 °C) and pressures (180, 200, and 220 bar) for 1.5 h.

An investigation was conducted into the stability based on the peroxide value of oil extracted from tray-dried bee brood, utilizing supercritical CO_2_ under various conditions. All oil samples initially exhibited comparable peroxide values, ranging from 8.79 to 9.82 meqO_2_/kg of oil. As depicted in Figure 2, the higher peroxide values observed over time indicated a decrease in oxidative stability. Notably, oil stored at 55 °C exhibited a higher peroxide value compared to oil stored at 25 °C. This highlighted the influence of storage temperature on the oxidative stability of the oil extracted from tray-dried bee brood.

Oil extracted from various insect types using supercritical CO_2_ may have different peroxide values. However, during storage, the peroxide value of extracted insect oil was found to increase with longer storage time and higher temperatures. For instance, Muangrat and Pannasai [17] observed a significant increase in peroxide value in oil extracted from black soldier fly larvae with higher storage temperatures (25 to 55 °C) and longer storage times (0 to 90 days). Similarly, Kim and Oh [22] found that peroxide values increased with storage time in various oil samples, including Tenebrio molitor larval oil, olive oil, and oleogels.

### 3.5. Total Phenolic Compound Content, Total Flavonoid Content, and Antioxidant Activities

This study investigated the content of total phenolic compounds in oil extracted from bee brood under various extraction conditions, as presented in Table 4. The average content of total phenolic compounds ranged from 56.13 to 69.65 μg of gallic acid equivalent per gram of oil. Furthermore, the results suggested that with an increase in extraction temperature, there was a tendency for the average total phenolic compound content to increase. Additionally, at any given extraction temperature, increasing the extraction pressure from 180 to 220 bar could potentially lead to an increase in the total phenolic compound content. However, this increase was not found to be statistically significant.

Although the increase in the average amount of total phenolic compounds was statistically insignificant, higher extraction pressure demonstrated the potential for increasing their content at a constant temperature. This suggests a positive relationship between extraction pressure and phenolic compound solubility in supercritical CO_2_. The improved solubility of phenolic compounds is attributed to the enhanced solvating power of CO_2_ at higher pressures, which better facilitates their dissolution. Meanwhile, higher extraction temperature aided in enhancing extraction effectiveness by reducing the viscosity of the supercritical CO_2_ solvent, facilitating improved penetration into the tray-dried bee brood samples and thereby potentially increasing the total phenolic compound content [45].

The antioxidant activity values, assessed through DPPH, ABTS, and FRAP assays, exhibited a correlation with the results of the total phenolic compound analysis. It was observed that higher extraction temperatures could potentially enhance extraction efficiency by reducing solvent viscosity. This could facilitate improved penetration into the samples, thus enhancing the extraction of antioxidant compounds [45]. The results were similar to those of Muangrat and Pannasai [17], who found that oil extracted from black soldier fly larvae using supercritical CO_2_ showed higher amounts of total phenolic compounds and increased antioxidant activity when the extraction temperature was increased from 45 to 55 °C, with an extraction time of 1 to 5 h and a constant pressure of 220 bar.

At an extraction temperature of 40 °C, increasing the extraction pressure from 180 to 200 bar increased total flavonoid content; however, a further increase in extraction pressure to 220 bar led to a decrease in total flavonoid content. Subsequent experiments involved increasing the extraction temperature to 50 °C, indicating a continuous decrease in total flavonoids until reaching 60 °C, where an increase in total flavonoid content was observed. However, at 60 °C, raising the pressure resulted in a reduction in total flavonoids. The fluctuating trend in total flavonoid content indicated that both extraction temperature and pressure significantly influence changes in total flavonoid content. Changes in extraction pressure and temperature can influence both the solubility of flavonoids and their total yield in supercritical CO_2_.

Furthermore, based on the experimental results presented in Table 3, the peroxide value is a measure of the extent of oxidation in oils and fats, indicating the amount of peroxides present. High peroxide values suggest increased lipid oxidation, which can lead to rancidity and reduced product quality. On the other hand, total phenolic content and total flavonoid content are measures of antioxidant compounds in a substance. These compounds can inhibit oxidation and help maintain the stability of oils and fats. The relationship between peroxide value changes and total phenolic content or flavonoid content lies in their roles in oxidative processes. Higher levels of phenolic compounds and flavonoids in oils and fats can act as antioxidants, scavenging free radicals and preventing the formation of peroxides [46,47,48,49]. Therefore, oils with higher total phenolic content or flavonoid content are likely to exhibit lower peroxide values, indicating better resistance to oxidation and higher quality. In summary, there is a correlation between peroxide value changes and total phenolic content or flavonoid content in oils and fats. Higher levels of these antioxidant compounds are associated with lower peroxide values, suggesting increased stability and quality of the product [46,47,48,49].

Increasing the extraction pressure at a low extraction temperature of 40 °C led to a higher yield of carotenoids. This was attributed to the enhanced solubility of carotenoids in the supercritical CO_2_ solvent at higher extraction pressures. In contrast, carotenoid extraction decreased at higher extraction temperatures (50 °C and 60 °C). Interestingly, when combined with higher extraction pressure at these elevated temperatures, there was a tendency for carotenoid levels to increase. The findings indicated that extraction temperature had a more significant impact on reducing carotenoids than higher extraction pressure (Table 4).

To evaluate the effectiveness of high-pressure extraction, this study used tray-dried bee broods and subjected them to supercritical CO_2_ extraction at a higher pressure of 600 bar for 1 h. The results showed that extraction at 600 bar yielded 2.46 to 7.35 times more crude oil than at lower pressures (180–220 bar) at the same extraction temperature, resulting in reduced lipid content in the defatted bee brood meal (as illustrated in Table 2 and Table 5). High extraction pressure reduces the impact of temperature changes and enhances the ability of supercritical CO_2_ to dissolve matrix components [50], resulting in a significant increase in extracted oil yield.

Furthermore, Table 5 provides the percentages of moisture content, protein, lipid, ash, crude fiber, and carbohydrates in defatted bee brood meal obtained after the extraction process using supercritical CO_2_. A higher quantity of extracted oil was obtained through increased extraction, leading to a reduced residual amount of defatted bee brood meal from the supercritical CO_2_ extraction process. Additionally, it is evident that the post-extraction defatted bee brood meal possesses a noteworthy protein content, presenting itself as a promising protein source worthy of further development.

Table 5 shows the acid, iodine, saponification, and peroxide values in the extracted oil. As the extraction temperature increased from 40 °C to 60 °C, there was a noticeable decrease in the acid, iodine, and saponification values of the extracted oil. Furthermore, elevating the extraction temperature, coupled with a high extraction pressure of 600 bar, resulted in an increased peroxide value for the extracted oil. Additionally, it is worth highlighting that raising the extraction pressure to 600 bar across extraction temperatures of 40 °C, 50 °C, and 60 °C led to a notable decrease in the acid value compared to lower extraction pressures (180–220 bar) within the same temperature range (Table 2). Regarding the iodine value of bee brood oil extracted at a high pressure of 600 bar, as indicated in Table 5, it closely resembled the iodine value obtained at an extraction pressure of 220 bar and extraction temperature of 40 °C, as well as the values achieved at an extraction pressure of 200 bar and extraction temperature of 50 °C, and at an extraction pressure ranging from 180 to 200 bar with an extraction temperature of 60 °C, as shown in Table 3. The saponification values of bee brood oil extracted under a pressure of 600 bar ranged from 180.89 to 190.52 mgKOH/g oil, showing a saponification value close to that obtained from extraction under a pressure of 220 bar and at a temperature of 40 °C. The peroxide value of bee brood oil extracted under high-pressure conditions (600 bar) was noted to be lower than that extracted under lower pressure (180–220 bar) at extraction temperatures ranging from 40–50 °C. However, elevating the extraction temperature to 60 °C at 600 bar pressure resulted in an increase in peroxide value, surpassing that of oil extracted under lower pressure. This increase can be attributed to the accelerated oxidation process at higher temperatures, leading to a higher peroxide value when employing both higher extraction pressure and temperature.

Table 5 presents the antioxidation capabilities assessed through DPPH, ABTS, and FRAP assays, alongside the total phenolic compound content and total flavonoid content. However, no statistically significant difference was observed in the antioxidant capacity and total phenolic compound content between the oil extracted at temperatures of 50 and 60 °C, both conducted at an extraction pressure of 600 bar for 1 h. Nevertheless, when the extraction pressure was increased to 600 bar, higher temperatures led to a notable increase in phenolic compound content in the extracted oil, consequently enhancing its resistance to oxidation. Moreover, these values were markedly higher compared to the oil extracted from tray-dried bee brood at an extraction temperature of 40 ºC under the same extraction pressure conditions.

The total flavonoid content decreased compared to lower extraction pressures (180–220 bar) at the same extraction temperature. However, there were no significant differences in the total flavonoid content of oils extracted from tray-dried bee brood at the higher extraction pressure of 600 bar (as shown in Table 5).

### 3.6. Identification and Quantification of Phenolic Compounds 

Tray-dried bee brood and bee brood oil obtained through supercritical CO_2_ extraction (conducted at 50 °C and an extraction pressure of 600 bar for 1 h) were selected to identify and quantify phenolic compounds. The chemical screening was performed by thin-layer chromatography (TLC), comparing the samples with more than 15 authentic compounds such as quercetin, gallic acid, lutein, kaempferol, lupeol, eugenol, betulinic acid, etc. The TLC chromatogram indicated that quercetin was a major compound in both samples. Therefore, precise identification was carried out using HPLC. The results revealed that both samples predominantly featured quercetin among the analyzed phenolic compounds. Quercetin content was 296.9 µg/mL (0.03% *w*/*w*) for tray-dried bee brood and 203.6 µg/mL (0.02% *w*/*w*) for the extracted oil. These findings are visually depicted in Supplemental Figures S3 and S4.

### 3.7. Fatty Acid Composition of Extracted Oil 

Considering the results of low-pressure extraction (Table 6), the investigation revealed the presence of significant fatty acids in the extracted oils, such as palmitic acid, oleic acid, linoleic acid, stearic acid, myristic acid, eicosadienoic acid, and docosadienoic acid. The information on the ratio of polyunsaturated fatty acids (PUFAs) to saturated fatty acids (SFAs), the index of atherogenicity (IA), the index of thrombogenicity (IT), and the hypocholesterolemic/hypercholesterolemic (H/H) ratio offered insight on possible nutritional and cardiometabolic health implications associated with the consumption of bee brood oil.

Table 6 shows that oils extracted from tray-dried bee brood contain varying proportions of saturated fatty acids (SFAs) (45.77–55.76 g/100 g), monounsaturated fatty acids (MUFAs) (30.33–37.94 g/100 g), and polyunsaturated fatty acids (PUFAs) (8.49–23.63 g/100 g). There are reports on the fatty acid composition of bee brood by Guiné et al. [2] and Finke [5] that align with these findings. Guiné et al. [2] reported that bee brood was primarily composed of SFAs (palmitic acid and stearic acid) and MUFAs (oleic acid), with a small amount of PUFAs such as linoleic acid. Similarly, Finke [5] found that the fatty acid profile of honeybee brood mainly consisted of oleic acid and palmitic acid, with most fat being SFAs (51.8%) and MUFAs (46.2%), and only a minor portion being PUFAs (2.0%). It appears that the supercritical CO_2_ extraction method can extract oil from tray-dried bee brood while maintaining a similar main fatty acid composition as detected in the bee brood.

The PUFAs/SFAs ratio, a common gauge for assessing the cardiovascular health implications of dietary choices, indicates that dietary PUFAs may decrease low-density lipoprotein cholesterol (LDL-C) and overall serum cholesterol levels, while SFAs may contribute to elevated serum cholesterol [24]. The PUFAs/SFAs ratios in oils extracted from tray-dried bee brood (ranging from 0.20 to 0.52) were notably higher than the PUFAs/SFAs ratio of palm stearin (0.13) but lower than that of sunflower oil (4.75–4.94) compared to data presented by Chen and Liu [24]. The suggested recommendation for the PUFA/SFA ratio was to exceed 0.4, aiming to diminish the potential risks associated with cardiovascular, autoimmune, and various chronic diseases [26]. Moreover, a reduced PUFAs/SFAs ratio signifies an elevated presence of dietary saturated fatty acids, a recognized major risk factor for cardiovascular disease. In this study, the ratio fell below the minimum recommended threshold for some tray-dried bee brood oils. It was interesting to observe that none of the oil extracted at 600 bar had a PUFAs/SFAs ratio of below 0.4, regardless of extraction temperature, while oils using a lower extraction pressure of 180 bar reached the recommended value (Table 6 and Table 7). The n-6/n-3 ratio identified in the oils extracted from tray-dried bee brood ranged from 0.92 to 8.42. 

Lipids with a lower index of atherogenicity (IA) or index of thrombogenicity (IT) are generally considered nutritionally superior, implying a reduced risk of contributing to coronary heart diseases [24]. According to Krešić et al. [51], recommended values for IA (<1) and IT (<1) suggest positive health benefits from the product. The index of atherogenicity (IA), a measure of the lipid’s anti-atherogenic potential [24,52], revealed that the IA values of oils derived from tray-dried bee brood (1.15–1.43) surpassed those found in edible plant oils such as camelina oil (0.05–0.07) and sunflower oil (0.09–0.11) [24]. The index of thrombogenicity (IT), indicating the thrombogenic potential of fatty acids and assessing the risk of clot formation in blood vessels, displayed a range of 1.50 to 2.28 for the oils extracted from tray-dried bee brood. Significantly, these oils exhibited higher IT values compared to plant oils such as soybean oil (0.40) [53] and camelina oil (0.10) [24]. Notably, coconut oil, with an IA value of 13.63, was identified as a highly atherogenic food. In contrast, raw mackerel, olive oil, and sunflower oil, with IA values of 0.28, 0.14, and 0.07, respectively, have been reported as foods with lower atherogenic potential [42,54]. However, this study observed higher IA and IT indices in all extracted tray-dried bee brood oils.

The hypocholesterolemic/hypercholesterolemic (H/H) ratio, originally designed to investigate the influence of fatty acid composition on blood cholesterol levels and cardiovascular disease risk [55,56], is indicative of its impact on human health [24]. Higher H/H values are typically viewed as more favorable for well-being [24]. Oils extracted under conditions of 180 bar and 40 °C displayed the highest H/H ratio of 1.35. Nonetheless, overall, the H/H ratios of tray-dried bee brood oils (0.90–1.35) were notably lower than the values observed in camelina oil (11.2–15.0) in the study conducted by Chen and Liu [24]. 

Table 7 displays the content of key fatty acids, including palmitic acid, oleic acid, stearic acid, linoleic acid, and myristic acid, of tray-dried bee brood oils, extracted at higher pressures. The oils from tray-dried bee brood varied in SFAs (51.51–57.46 g/100 g), MUFAs (34.85–38.99 g/100 g), and PUFAs (3.54–13.64 g/100 g). PUFAs/SFAs ratios ranged from 0.06 to 0.26. The n-6/n-3 ratio was 2.11 to 3.33. Tray-dried bee brood oils had IA values of 1.28 to 1.36. The IT range for tray-dried bee brood oils was 1.91 to 2.54, and the H/H ratio ranged from 0.89 to 1.09. The data presented in Table 7 indicate that employing supercritical CO_2_ extraction for tray-dried bee brood oils at a higher pressure of 600 bar led to an apparent increase in SFAs and MUFAs, along with a concurrent decline in PUFAs. Conversely, this process exhibited only a marginal impact on IA value, IT value, n-6/n-3 ratio, and H/H ratio compared to extractions conducted at lower pressures ranging from 180 to 220 bar. 

The nutritional indicators in Table 6 and Table 7 provide an initial assessment for incorporating tray-dried bee brood oil into various food industry applications. Guiné et al. [2] highlighted that honeybee brood, valued for its high nutritional content, is available in specialized markets and among ethnic communities with traditional consumption practices. However, insect-based products often face rejection due to neophobia and disgust. Incorporating honeybee brood into Western diets may be facilitated by culinary chefs using traditional recipes. Our study demonstrates that supercritical CO_2_ extraction of bee brood produces oil that addresses these challenges, making it a viable ingredient for functional foods and cosmetics. Rich in essential fatty acids (Table 6 and Table 7) and potent antioxidants (Table 4 and Table 5), this oil supports cardiovascular health, brain function, and inflammation reduction. MUFAs enhance cholesterol levels, while antioxidants protect cells from oxidative damage, support the immune system, and improve skin health by reducing signs of aging and aiding healing. Therefore, extracted bee brood oil is likely to attract attention and may hold significant potential for development as both a dietary supplement and a skincare ingredient.

### 3.8. Amino Acid Composition

The tray-dried bee brood sample and defatted bee brood meal, following extraction with supercritical CO_2_, exhibited similar profiles of important amino acids. As shown in Table 8, it can be concluded that the supercritical CO_2_ extraction process applied to obtain tray-dried bee brood oil had minimal impact on the amino acid content of the defatted bee brood meal after extraction under a low extraction pressure of 220 bar. The amino acids present in the tray-dried bee brood sample before oil extraction and the defatted bee brood after extraction with supercritical CO_2_ included glutamic acid, aspartic acid, leucine, lysine, proline, arginine, tyrosine, serine, alanine, threonine, isoleucine, glycine, and phenylalanine, respectively. Notably, glutamic acid was the most predominant among them. This study’s conclusions are similar to those of Guiné et al. [2] and Finke [5], who reported that leucine, lysine, and aromatic amino acids are the most prevalent essential amino acids, while glutamic acid is the most abundant non-essential amino acid.

When utilizing supercritical CO_2_ extraction to extract oil from tray-dried bee brood at a higher extraction pressure of 600 bar for 1 h, the defatted bee brood demonstrated diminished amino acid levels compared to extraction at a lower pressure (200 bar, 1.5 h). Remarkably, hydroxylysine was detected in low amounts, whereas L-cystine and hydroxyproline were found in higher quantities. Valine was not detected during the examination of the amino acid profile. The defatted tray-dried bee brood meal, obtained through supercritical CO_2_ extraction at higher pressures, demonstrated reduced amino acid content compared to extraction performed at lower pressures, as detailed in Table 8. Ideally, residues from supercritical fluid extraction should maintain consistent chemical compositions and nutritional properties. However, variations in extraction parameters, such as pressure, temperature, and time, may result in protein denaturation and coextraction of undesirable protein compounds, ultimately leading to reduced protein content in defatted samples and degradation of amino acids, resulting in the loss of amino acids [57]. 

Further investigation is needed to determine the optimal extraction pressure in the supercritical CO_2_ extraction process, ensuring the highest oil yield from bee broods while retaining elevated amino acid levels in defatted bee brood meal. The study findings confirmed that even after supercritical CO_2_ extraction, the defatted tray-dried bee brood meal retained substantial levels of amino acids. Furthermore, this study revealed that the amino acid content of the samples remained unaffected when employing the supercritical CO_2_ extraction method at low pressure, in contrast to higher pressure conditions. Consequently, it can be concluded that defatted tray-dried bee brood meal is a noteworthy source of essential amino acids.

## 4. Conclusions

The application of the supercritical CO_2_ extraction process on tray-dried bee brood showcased significantly enhanced efficiency, resulting in approximately 3–4 times higher oil content extraction when conducted at elevated pressure at 600 bar. This contrasted with lower pressures in the 180–220 bar range, maintaining the same applied extraction temperatures (40–60 °C). The elevation of extraction pressure to 600 bar within this temperature range increased total phenolic compounds, while total flavonoids decreased. Notably, the oil extracted from tray-dried bee brood exhibited antioxidation activities (DPPH, ABTS, and FRAP assays), which were mostly due to quercetin, the main phenolic compound. Supercritical CO_2_ extraction at elevated pressure and temperature led to a decrease in acid value, iodine value, and saponification value. However, it concurrently resulted in a higher peroxide value, which remained below 12 meqO_2_/kg of oil. The oil extracted displayed diverse fatty acid compositions depending on extraction conditions. At temperatures ranging from 40–60 °C and pressures of 180–220 bar, the primary fatty acids included palmitic acid (36.33–44.91%), oleic acid (29.55–37.32%), linoleic acid (2.23–11.23%), stearic acid (5.09–6.82%), myristic acid (3.17–3.83%), eicosadienoic acid (1.93–6.46%), and docosadienoic acid (0.56–4.69%). When the extraction pressure was increased to 600 bar under the same temperature conditions, the predominant fatty acids remained palmitic acid (40.43–44.09%) and oleic acid (34.40–38.54%), with higher levels of stearic acid (5.09–6.82%), lower levels of myristic acid (2.88–3.45%), and reduced linoleic acid (1.29–5.75%) and docosadienoic acid (0.68–2.26%). Eicosadienoic acid was not detected under these conditions. Following defatting through supercritical CO_2_ extraction, the bee brood maintained significant concentrations of essential and non-essential amino acids, underscoring its potential as a valuable protein source. However, the next phase of our research will explore various drying methods affecting the extracted bee brood oil and its physicochemical properties, as well as investigate the bioactive components of the extracted bee brood oil in greater detail.

## Figures and Tables

**Table 1:** Percentage of moisture content, protein, lipid, ash, crude fiber, and carbohydrate of bee brood and tray-dried bee brood before supercritical CO_2_ extraction.

Sample	Moisture Content (%)	Protein (%)	Lipid (%)	Ash (%)	Crude Fiber (%)	Carbohydrate (%)
Bee brood	71.59 ± 0.52 ^a^	10.22 ± 0.18 ^b^	1.57 ± 0.38 ^b^	2.30 ± 0.04 ^b^	12.17 ± 0.71 ^a^	2.15 ± 0.97 ^b^
Tray-dried bee brood	2.27 ± 0.05 ^b^	41.72 ± 0.71 ^a^	34.64 ± 0.48 ^a^	3.25 ± 0.06 ^a^	3.37 ± 0.08 ^b^	15.88 ± 0.60 ^a^

Note: Means in the same column with different letters are considered statistically significantly different at the 95 percent confidence level (*p* < 0.05) based on the *t*-test analysis.

**Table 2:** Percentage yield and chemical properties (acid value, iodine value, saponification value, and peroxide value) of oil extracted from tray-dried bee brood using supercritical CO_2_ extraction at various extraction temperatures (40 °C, 50 °C, and 60 °C) and extraction pressures (180, 200, and 220 bar).

Extraction Conditions		%Crude Oil	Acid Value (mg KOH/g Oil)	Iodine Value (g I_2_/100 g Oil)	Saponification Value (mg KOH/g Oil)	Peroxide Value (meqO_2_/kg Oil)
Temperature (°C)	Pressure (Bar)					
40	180	6.14 ± 0.82 ^c^	49.32 ± 0.76 ^a^	39.34 ± 0.64 ^a^	292.40 ± 1.10 ^a^	9.14 ± 0.46 ^ab^
40	200	7.46 ± 1.57 ^abc^	33.72 ± 0.82 ^c^	30.54 ± 0.44 ^b^	277.40 ± 0.51 ^b^	9.27 ± 0.52 ^ab^
40	220	8.67 ± 1.35 ^a^	37.89 ± 0.51 ^b^	21.22 ± 0.65 ^d^	188.60 ± 1.70 ^d^	8.79 ± 0.17 ^b^
50	180	4.21 ± 0.60 ^d^	38.43 ± 0.65 ^b^	25.26 ± 0.39 ^c^	209.57 ± 1.19 ^c^	9.27 ± 0.48 ^ab^
50	200	6.86 ± 1.39 ^bc^	33.23 ± 0.49 ^c^	20.97 ± 0.40 ^d^	171.48 ± 1.36 ^e^	9.60 ± 0.30 ^a^
50	220	8.05 ± 0.69 ^ab^	27.58 ± 0.90 ^e^	16.04 ± 0.88 ^e^	157.62 ± 0.91 ^f^	9.34 ± 0.16 ^ab^
60	180	3.45 ± 0.43 ^d^	29.70 ± 0.94 ^d^	11.89 ± 0.64 ^f^	130.66 ± 0.79 ^g^	9.48 ± 0.04 ^ab^
60	200	4.21 ± 0.56 ^d^	27.59 ± 0.90 ^e^	10.83 ± 0.81 ^g^	115.46 ± 1.38 ^h^	9.82 ± 0.26 ^a^
60	220	6.22 ± 1.26 ^c^	25.46 ± 0.65 ^f^	7.55 ± 0.19 ^h^	98.91 ± 1.24 ^i^	9.63 ± 0.20 ^a^

Note: Means in the same column with different letters are considered statistically significantly different at the 95 percent confidence level (*p* < 0.05). Each condition of the supercritical CO_2_ extraction was performed for 1.5 h.

**Table 3:** Composition of defatted tray-dried bee brood meal obtained through supercritical CO_2_ extraction.

Extraction Conditions		Moisture Content (%)	Protein (%)	Lipid (%)	Crude Oil (%)	Ash (%)	Carbohydrate (%)
Temeprature (°C)	Pressure (Bar)						
40	180	2.22 ± 0.04 ^c^	43.83 ± 0.16 ^ab^	31.00 ± 0.99 ^bc^	2.95 ± 0.38 ^ab^	0.26 ± 0.08 ^d^	19.75 ± 1.14 ^ab^
40	200	2.14 ± 0.05 ^c^	43.78 ± 1.27 ^ab^	30.49 ± 1.66 ^bc^	2.66 ± 0.26 ^bc^	0.97 ± 0.10 ^bc^	19.97 ± 3.15 ^a^
40	220	2.36 ± 0.15 ^c^	45.29 ± 0.72 ^a^	30.22 ± 1.46 ^c^	2.58 ± 0.35 ^bc^	0.96 ± 0.49 ^bc^	18.59 ± 1.28 ^ab^
50	180	2.03 ± 0.14 ^c^	42.70 ± 0.55 ^b^	32.74 ± 1.41 ^abc^	2.73 ± 0.01 ^bc^	1.09 ± 0.08 ^b^	18.71 ± 1.85 ^ab^
50	200	2.02 ± 0.26 ^c^	43.40 ± 0.53 ^b^	31.73 ± 0.43 ^bc^	2.55 ± 0.07 ^bc^	0.71 ± 0.07 ^c^	19.59 ± 0.38 ^ab^
50	220	6.84 ± 0.21 ^b^	43.16 ± 0.70 ^b^	31.10 ± 1.83 ^bc^	2.50 ± 0.02 ^c^	2.23 ± 0.06 ^a^	14.18 ± 1.79 ^c^
60	180	7.57 ± 0.32 ^a^	42.33 ± 0.76 ^b^	34.44 ± 0.45 ^a^	2.62 ± 0.05 ^bc^	2.12 ± 0.19 ^a^	10.92 ± 0.28 ^d^
60	200	7.49 ± 0.26 ^a^	42.43 ± 1.56 ^b^	34.18 ± 1.65 ^a^	2.79 ± 0.15 ^abc^	2.27 ± 0.06 ^a^	10.83 ± 0.51 ^d^
60	220	1.62 ± 0.16 ^d^	43.17 ± 1.29 ^b^	32.79 ± 1.10 ^ab^	3.12 ± 0.33 ^a^	2.26 ± 0.02 ^a^	17.05 ± 0.84 ^b^

Note: Mean ± standard deviation values with different lowercase letters in the same column are significantly different at *p* < 0.05. Each condition of the supercritical CO_2_ extraction was performed for 1.5 h.

**Table 4:** Antioxidative activities (DPPH, ABTS, and FRAP assays), total phenolic content, total flavonoid content, and carotenoid content of extracted oil obtained from tray-dried bee brood using supercritical CO_2_.

Extraction Conditions		DPPH (μmol TE/g Oil)	ABTS (μmol TE/g Oil) ^ns^	FRAP (μmol TE/g Oil)	TPC (μg GAE/g Oil)	TFC (μg QE/g Oil)	TCC (μg βE/g Oil)
Temperature (°C)	Pressure (Bar)						
40	180	2.78 ± 0.12 ^b^	1.32 ± 0.20	1.33 ± 0.08 ^d^	56.13 ± 3.13 ^c^	3.08 ± 0.22 ^d^	1.52 ± 0.07 ^f^
40	200	2.34 ± 0.16 ^c^	1.31 ± 0.13	1.41 ± 0.18 ^d^	62.24 ± 3.08 ^abc^	10.45 ± 0.30 ^a^	2.37 ± 0.08 ^c^
40	220	2.19 ± 0.26 ^cd^	1.35 ± 0.27	1.60 ± 0.25 ^d^	62.63 ± 1.26 ^abc^	3.62 ± 0.23 ^c^	7.32 ± 0.07 ^a^
50	180	2.07 ± 0.07 ^d^	1.25 ± 0.20	2.54 ± 0.10 ^c^	63.74 ± 1.17 ^ab^	3.21 ± 0.30 ^d^	2.57 ± 0.15 ^b^
50	200	1.18 ± 0.09 ^f^	1.38 ± 0.19	2.72 ± 0.08 ^bc^	66.14 ± 5.25 ^ab^	0.81 ± 0.09 ^f^	1.74 ± 0.16 ^e^
50	220	3.06 ± 0.04 ^a^	1.39 ± 0.08	2.87 ± 0.10 ^ab^	61.04 ± 2.45 ^bc^	0.21 ± 0.05 ^g^	2.59 ± 0.11 ^b^
60	180	2.34 ± 0.15 ^c^	1.60 ± 0.16	3.19 ± 0.38 ^a^	66.47 ± 2.15 ^ab^	10.53 ± 0.20 ^a^	1.05 ± 0.08 ^g^
60	200	1.40 ± 0.03 ^ef^	1.53 ± 0.05	2.91 ± 0.30 ^ab^	67.05 ± 6.70 ^ab^	9.30 ± 0.19 ^b^	1.95 ± 0.07 ^d^
60	220	1.51 ± 0.09 ^e^	1.51 ± 0.06	2.98 ± 0.16 ^ab^	69.65 ± 0.62 ^a^	1.37 ± 0.22 ^e^	1.74 ± 0.16 ^e^

Note: Means in the same column with different letters are considered statistically significantly different at the 95 percent confidence level (*p* < 0.05). ns: not significant. TPC: Total phenolic compound content. TFC: Total flavonoid content. TCC: Total carotenoid content. GAE: gallic acid equivalent. QE: quercetin equivalent. TE: Trolox equivalent. βE: β-carotene equivalents. Each condition of the supercritical CO_2_ extraction was performed for 1.5 h.

**Table 5:** Qualities of extracted oil and defatted tray-dried bee brood meal obtained through the utilization of supercritical CO_2_ at 40–60 °C, 600 bar for 1 h.

Qualities of Extracted Oil	Extraction Temperatures		
	40 °C	50 °C	60 °C
% Crude oil ^ns^	21.41 ± 1.07	21.50 ± 2.38	25.37 ± 1.52
Acid value (mg KOH/g)	32.51 ± 0.31 ^a^	20.97 ± 0.61 ^b^	10.75 ± 0.23 ^c^
Iodine value (g I_2_/100 g)	23.06 ± 0.68 ^a^	12.52 ± 0.65 ^b^	10.34 ± 0.35 ^c^
Saponification value (mg KOH/g) ^ns^	190.52 ± 2.12	181.79 ± 8.05	180.89 ± 2.48
Peroxide value (meqO_2_/kg)	7.28 ± 0.55 ^b^	8.95 ± 1.98 ^ab^	11.62 ± 0.21 ^a^
TPC (µg GAE/g oil)	67.87 ± 1.12 ^b^	71.58 ± 2.02 ^a^	71.05 ± 0.88 ^a^
TFC (µg QE/g oil) ^ns^	1.04 ± 0.19	0.85 ± 0.00	0.91 ± 0.11
DPPH **(**µmol TE/g oil)	2.11 ± 0.09 ^ab^	2.25 ± 0.07 ^a^	1.97 ± 0.05 ^b^
ABTS **(**µmol TE/g oil) ^ns^	0.41 ± 0.03	0.46 ± 0.05	0.41 ± 0.04
FRAP **(**µmol TE/g oil)	1.31 ± 0.02 ^b^	1.44 ± 0.09 ^a^	1.40 ± 0.11 ^ab^
**Defatted tray-dried bee brood meal**			
Moisture content (%)	6.19 ± 0.04 ^b^	6.45 ± 0.02 ^a^	6.41 ± 0.04 ^a^
Protein (%)	50.03 ± 1.01 ^b^	50.36 ± 0.58 ^b^	52.98 ± 0.90 ^a^
Lipid (%)	11.29 ± 0.26 ^a^	10.59 ± 0.15 ^a^	6.05 ± 0.60 ^b^
Crude fiber (%) ^ns^	2.70 ± 0.43	2.53 ± 0.27	2.54 ± 0.15
Ash (%) ^ns^	2.79 ± 0.12	2.86 ± 0.03	2.97 ± 0.22
Carbohydrate (%)	26.99 ± 0.26 ^b^	27.21 ± 0.91 ^b^	29.05 ± 1.26 ^a^

Note: Means in the same row with different letters are considered statistically significantly different at the 95 percent confidence level (*p* < 0.05). ns: not significant. TPC: Total phenolic compound content. TFC: Total flavonoid content. GAE: gallic acid equivalent. QE: quercetin equivalent. TE: Trolox equivalent.

**Table 6:** Fatty acid composition of tray-dried bee brood oil extracted by using supercritical CO_2_.

Fatty Acid Composition (g/100 g)	Tray-Dried Bee Brood Oil Extracted by Using Supercritical CO_2_								
	40 °C, 180 Bar	40 °C, 200 Bar	40 °C, 220 Bar	50 °C, 180 Bar	50 °C, 200 Bar	50 °C, 220 Bar	60 °C, 180 Bar	60 °C, 200 Bar	60 °C, 220 Bar
Butyric acid (C4:0)	ND	0.06 ± 0.00 ^cd^	0.05 ± 0.00 ^d^	0.09 ± 0.00 ^b^	0.16 ± 0.02 ^a^	0.07 ± 0.01 ^c^	ND	ND	ND
Caproic acid (C6:0)	ND	0.01 ± 0.00	ND	ND	ND	ND	ND	ND	ND
Caprylic acid (C8:0)	ND	0.01 ± 0.00	ND	ND	ND	ND	ND	ND	ND
Capric acid (C10:0)	ND	0.03 ± 0.00	ND	ND	ND	0.03 ± 0.00	0.04 ± 0.01	ND	ND
Lauric acid (C12:0)	0.26 ± 0.00 ^d^	0.33 ± 0.01 ^bcd^	0.33 ± 0.02 ^abcd^	0.33 ± 0.01 ^bcd^	0.30 ± 0.05 ^de^	0.37 ± 0.01 ^ab^	0.36 ± 0.03 ^abc^	0.38 ± 0.04 ^a^	0.32 ± 0.01 ^cd^
Myristic acid (C14:0) ^ns^	3.17 ± 0.06	3.50 ± 0.02	3.77 ± 0.01	3.66 ± 0.01	3.49 ± 0.07	3.82 ± 0.03	3.30 ± 0.03	3.62 ± 0.07	3.83 ± 0.03
Myristoleic acid (C14:1)	ND	0.03 ± 0.00	ND	ND	ND	ND	ND	ND	ND
Pentadecylic acid (C15:0)	ND	ND	ND	ND	ND	ND	ND	ND	ND
Pentadecenoic acid (cis-10) (C15:1)	0.05 ± 0.00	ND	ND	ND	ND	ND	ND	ND	ND
Palmitic acid (C16:0)	36.33 ± 0.44 ^e^	38.55 ± 0.02 ^c^	42.91 ± 0.13 ^b^	42.58 ± 0.03 ^b^	37.43 ± 0.10 ^d^	44.91 ± 0.89 ^a^	37.57 ± 0.16 ^d^	43.02 ± 0.09 ^b^	44.52 ± 0.06 ^a^
Palmitoleic acid (C16:1)	0.37 ± 0.03 ^c^	0.53 ± 0.01 ^bc^	0.52 ± 0.01 ^bc^	0.53 ± 0.03 ^bc^	0.47 ± 0.01 ^b^	0.52 ± 0.01 ^bc^	0.50 ± 0.06 ^bc^	0.56 ± 0.01 ^a^	0.53 ± 0.02 ^bc^
Margaric acid (C17:0)	ND	ND	ND	ND	ND	ND	ND	ND	ND
Heptadecenoic acid(cis-10) (C17:1)	ND	0.12 ± 0.01 ^c^	0.08 ± 0.00 ^e^	0.10 ± 0.00 ^d^	0.15 ± 0.02 ^b^	0.06 ± 0.00 ^e^	0.25 ± 0.01 ^a^	0.12 ± 0.00 ^c^	0.04 ± 0.01 ^f^
Stearic acid (C18:0)	5.75 ± 0.08 ^d^	6.07 ± 0.04 ^c^	6.73 ± 0.01 ^a^	6.30 ± 0.01 ^b^	5.64 ± 0.02 ^d^	6.82 ± 0.16 ^a^	5.09 ± 0.03 ^e^	5.95 ± 0.08 ^c^	6.73 ± 0.03 ^a^
Elaidic acid (C18:1 n9t)	0.40 ± 0.12 ^a^	0.17 ± 0.02 ^c^	0.14 ± 0.00 ^cd^	0.17 ± 0.01 ^c^	0.17 ± 0.02 ^c^	0.08 ± 0.03 ^de^	0.32 ± 0.05 ^b^	0.13 ± 0.00 ^cd^	0.05 ± 0.01 ^e^
Oleic acid (C18:1 n9c)	29.90 ± 0.60 ^g^	32.55 ± 0.02 ^e^	34.36 ± 0.08 ^c^	34.78 ± 0.04 ^c^	31.74 ± 0.11 ^f^	35.98 ± 0.81 ^b^	29.55 ± 0.11 ^g^	33.66 ± 0.00 ^d^	37.32 ± 0.04 ^a^
Linoleic acid (C18:2 n6c)	11.15 ± 0.15 ^a^	7.59 ± 0.01 ^c^	4.50 ± 0.10 ^f^	5.36 ± 0.03 ^e^	9.04 ± 0.05 ^b^	3.75 ± 0.06 ^g^	11.23 ± 0.04 ^a^	5.69 ± 0.06 ^d^	2.23 ± 0.02 ^h^
Eicosadienoic acid (C20:0)	ND	0.27 ± 0.00 ^a^	0.12 ± 0.01 ^cd^	0.11 ± 0.00 ^cd^	0.10 ± 0.01 ^d^	0.12 ± 0.02 ^cd^	0.18 ± 0.04 ^b^	0.14 ± 0.04 ^c^	0.13 ± 0.01 ^cd^
γ-Linolenic acid (C18:3 n6) ^ns^	ND	ND	ND	ND	ND	ND	ND	ND	ND
Gondoic acid (C20:1)	ND	ND	ND	ND	ND	ND	ND	ND	ND
α-Linolenic acid (C18:3 n3)	0.57 ± 0.07 ^e^	0.61 ± 0.04 ^de^	0.52 ± 0.01 ^e^	0.72 ± 0.01 ^c^	0.85 ± 0.10 ^b^	0.53 ± 0.05 ^e^	1.34 ± 0.05 ^a^	0.76 ± 0.06 ^c^	0.68 ± 0.01 ^cd^
Eicosadienoic acid (C20:2)	6.46 ± 0.12 ^a^	4.89 ± 0.03 ^c^	2.68 ± 0.08 ^d^	2.52 ± 0.02 ^e^	4.90 ± 0.04 ^c^	1.93 ± 0.17 ^f^	5.26 ± 0.11 ^b^	2.67 ± 0.01 ^de^	1.08 ± 0.03 ^g^
Behenic acid (C22:0)	0.25 ± 0.07 ^a^	0.24 ± 0.00 ^a^	0.03 ± 0.00 ^c^	0.03 ± 0.00 ^c^	0.07 ± 0.02 ^bc^	0.02 ± 0.00 ^c^	0.09 ± 0.02 ^b^	ND	0.21 ± 0.01 ^a^
Dihomo-γ-linolenic acid (C20:3 n6)	ND	ND	ND	ND	ND	ND	ND	ND	ND
Eicosatrienoic acid (C20:3 n3)	ND	ND	ND	ND	ND	ND	ND	ND	ND
Tricosylic acid (C23:0)	ND	ND	ND	ND	ND	ND	ND	ND	ND
Arachidonic acid (C20:4 n6)	ND	ND	ND	ND	ND	ND	ND	ND	ND
Docosadienoic acid (C22:2)	4.69 ± 0.77 ^a^	3.70 ± 0.02 ^b^	2.62 ± 0.03 ^d^	1.54 ± 0.02 ^e^	3.02 ± 0.00 ^cd^	1.26 ± 0.17 ^e^	3.37 ± 0.01 ^bc^	1.61 ± 0.02 ^e^	0.56 ± 0.01 ^f^
Eicosapentaenoic acid (C20:5 n3)	0.76 ± 0.01 ^g^	0.75 ± 0.02 ^g^	0.59 ± 0.01 ^h^	1.21 ± 0.03 ^e^	2.48 ± 0.03 ^a^	1.02 ± 0.05 ^f^	1.54 ± 0.06 ^d^	1.68 ± 0.02 ^c^	1.75 ± 0.02 ^b^
Nervonic acid (C24:1n9)	ND	ND	ND	ND	ND	ND	ND	ND	ND
**SFAs**	45.77 ± 0.50 ^g^	49.07 ± 0.02 ^e^	53.99 ± 0.13 ^c^	53.11 ± 0.02 ^d^	47.18 ± 0.21 ^f^	54.87 ± 1.15 ^b^	46.63 ± 0.14 ^f^	53.11 ± 0.11 ^d^	55.76 ± 0.03 ^a^
**MUFAs**	30.33 ± 0.55 ^g^	33.39 ± 0.02 ^e^	35.11 ± 0.10 ^c^	35.54 ± 0.05 ^c^	32.53 ± 0.14 ^f^	36.64 ± 0.84 ^b^	30.63 ± 0.03 ^g^	34.48 ± 0.01 ^d^	37.94 ± 0.02 ^a^
**PUFAs**	23.63 ± 0.94 ^a^	17.54 ± 0.02 ^d^	10.91 ± 0.22 ^f^	11.35 ± 0.07 ^f^	20.29 ± 0.11 ^c^	8.49 ± 0.32 ^g^	22.74 ± 0.14 ^b^	12.41 ± 0.10 ^e^	6.30 ± 0.05 ^h^
**PUFAs/SFAs**	0.52 ± 0.03 ^a^	0.36 ± 0.00 ^d^	0.20 ± 0.00 ^f^	0.21 ± 0.00 ^f^	0.43 ± 0.00 ^c^	0.15 ± 0.01 ^g^	0.49 ± 0.00 ^b^	0.23 ± 0.00 ^e^	0.11 ± 0.00 ^h^
**n-6/n-3**	8.42 ± 0.56 ^a^	5.58 ± 0.07 ^b^	4.05 ± 0.03 ^c^	2.78 ± 0.02 ^d^	2.72 ± 0.11 ^de^	2.41 ± 0.04 ^ef^	3.90 ± 0.01 ^c^	2.33 ± 0.05 ^f^	0.92 ± 0.00 ^g^
**IA**	1.15 ± 0.01 ^c^	1.25 ± 0.00 ^bc^	1.43 ± 0.00 ^a^	1.34 ± 0.00 ^ab^	1.15 ± 0.01 ^c^	1.33 ± 0.22 ^ab^	1.14 ± 0.01 ^c^	1.36 ± 0.01 ^ab^	1.41 ± 0.00 ^b^
**IT**	1.82 ± 0.01 ^e^	1.94 ± 0.01 ^cd^	2.28 ± 0.01 ^a^	1.98 ± 0.01 ^c^	1.50 ± 0.02 ^f^	2.15 ± 0.10 ^b^	1.54 ± 0.01 ^f^	1.89 ± 0.02 ^d^	1.94 ± 0.00 ^cd^
**H/H ratio**	1.35 ± 0.03 ^a^	1.18 ± 0.00 ^c^	0.96 ± 0.01 ^de^	0.99 ± 0.00 ^d^	1.26 ± 0.01 ^b^	0.93 ± 0.05 ^ef^	1.27 ± 0.01 ^b^	0.98 ± 0.00 ^d^	0.90 ± 0.00 ^f^

Note: Under various conditions, the supercritical CO_2_ extraction process consistently necessitated an extraction time of 1.5 h. Means in the same row with different letters are considered statistically significantly different at the 95 percent confidence level (*p* < 0.05). ND: not detected; ns: not significant.

**Table 7:** Fatty acid composition of oil obtained from tray-dried bee brood, extracted using supercritical CO_2_.

Fatty Acid Composition (g/100 g)	Oil Obtained from Tray-Dried Bee Brood, Extracted Using Supercritical CO_2_		
	40 °C, 600 Bar, 1 h	50 °C, 600 Bar, 1 h	60 °C, 600 Bar, 1 h
Butyric acid (C4:0)	ND	ND	ND
Caproic acid (C6:0)	ND	ND	ND
Caprylic acid (C8:0)	ND	ND	ND
Capric acid (C10:0)	ND	ND	ND
Lauric acid (C12:0)	0.17 ± 0.01	ND	0.27 ± 0.02
Myristic acid (C14:0)	2.88 ± 0.02 ^c^	3.18 ± 0.03 ^b^	3.45 ± 0.07 ^a^
Myristoleic acid (C14:1)	ND	ND	ND
Pentadecylic acid (C15:0)	ND	ND	ND
Pentadecenoic acid (cis-10) (C15:1)	ND	ND	ND
Palmitic acid (C16:0)	44.09 ± 0.15 ^a^	42.63 ± 0.14 ^b^	40.43 ± 0.08 ^c^
Palmitoleic acid (C16:1) ^ns^	0.41 ± 0.01	0.44 ± 0.06	0.45 ± 0.01
Margaric acid (C17:0)	ND	ND	ND
Heptadecenoic acid(cis-10) (C17:1)	0.02 ± 0.00	0.07 ± 0.05	ND
Stearic acid (C18:0)	10.01 ± 0.03 ^a^	8.60 ± 0.09 ^b^	7.17 ± 0.15 ^c^
Elaidic acid (C18:1 n9t)	0.01 ± 0.01	0.54 ± 0.11	ND
Oleic acid (C18:1 n9c)	38.54 ± 0.09 ^a^	36.35 ± 0.15 ^b^	34.40 ± 0.15 ^c^
Linoleic acid (C18:2 n6c)	1.29 ± 0.03 ^c^	2.99 ± 0.02 ^b^	5.75 ± 0.03 ^a^
Eicosadienoic acid (C20:0)	0.23 ± 0.04	0.22 ± 0.04	ND
γ-Linolenic acid (C18:3 n6)	ND	ND	ND
Gondoic acid (C20:1)	ND	ND	ND
α-Linolenic acid (C18:3 n3)	0.34 ± 0.05	ND	0.55 ± 0.05
Eicosadienoic acid (C20:2)	0.96 ± 0.03 ^c^	2.11 ± 0.06 ^b^	3.61 ± 0.01 ^a^
Behenic acid (C22:0)	0.08 ± 0.02 ^b^	0.15 ± 0.03 ^a^	0.19 ± 0.02 ^a^
Dihomo-γ-linolenic acid (C20:3 n6)	ND	ND	ND
Eicosatrienoic acid (C20:3 n3)	ND	ND	ND
Tricosylic acid (C23:0)	ND	ND	ND
Arachidonic acid (C20:4 n6)	ND	ND	ND
Docosadienoic acid (C22:2)	0.68 ± 0.08 ^c^	1.60 ± 0.06 ^b^	2.26 ± 0.02 ^a^
Eicosapentaenoic acid (C20:5 n3)	0.28 ± 0.01 ^c^	0.90 ± 0.04 ^b^	1.47 ± 0.02 ^a^
Nervonic acid (C24:1n9)	ND	ND	ND
**SFAs**	57.46 ± 0.14 ^a^	54.78 ± 0.20 ^b^	51.51 ± 0.07 ^c^
**MUFAs**	38.99 ± 0.09 ^a^	37.38 ± 0.10 ^b^	34.85 ± 0.16 ^c^
**PUFAs**	3.54 ± 0.18 ^c^	7.59 ± 0.18 ^b^	13.64 ± 018 ^a^
**PUFAs/SFAs**	0.06 ± 0.00 ^c^	0.14 ± 0.00 ^b^	0.26 ± 0.00 ^a^
**n-6/n-3**	2.11 ± 0.15 ^c^	3.33 ± 0.14 ^a^	2.85 ± 0.07 ^b^
**IA**	1.36 ± 0.01 ^a^	1.34 ± 0.00 ^b^	1.28 ± 0.01 ^c^
**IT**	2.54 ± 0.03 ^a^	2.35 ± 0.01 ^b^	1.91 ± 0.00 ^c^
**H/H ratio**	0.89 ± 0.01 ^c^	0.96 ± 0.01 ^b^	1.09 ± 0.00 ^a^

Note: Means in the same row with different letters are considered statistically significantly different at the 95 percent confidence level (*p* < 0.05). ND: not detected. ns: not significant.

**Table 8:** Amino acid profile of the tray-dried bee brood sample and defatted bee brood meal after supercritical CO_2_ extraction.

Amino Acid Profile (mg/100 g)	Tray-Dried Bee Brood Sample	Defatted Tray-Dried Bee Brood Meal after Supercritical CO_2_ Extraction at 50 °C, 220 Bar, 1.5 h	Defatted Tray-Dried Bee Brood Meal after Supercritical CO_2_ Extraction at 50 °C, 600 Bar, 1 h
Alanine	2471.13 ± 1.57 ^a^	2397.86 ± 16.38 ^b^	1559.52 ± 7.56 ^c^
Arginine	2879.96 ± 104.09 ^a^	2839.02 ± 44.96 ^a^	1760.73 ± 36.34 ^b^
Aspartic acid	5557.26 ± 14.59 ^a^	5449.11 ± 19.74 ^b^	3070.62 ± 35.76 ^c^
L-Cystine	166.87 ± 0.47 ^b^	180.71 ± 0.68 ^b^	1406.06 ± 312.71 ^a^
Glutamic acid	8143.65 ± 83.23 ^a^	7996.52 ± 62.42 ^a^	4747.52 ± 203.39 ^b^
Glycine	2055.04 ± 3.18 ^a^	1990.11 ± 10.61 ^b^	1293.27 ± 0.25 ^c^
Histidine	1093.49 ± 6.03 ^a^	1077.59 ± 10.97 ^a^	670.29 ± 2.63 ^b^
Hydroxylysine	ND	ND	25.02 ± 0.50
Hydroxyproline	ND	ND	5299.62 ± 125.18
Isoleucine	2318.10 ± 0.46 ^a^	2211.53 ± 15.77 ^b^	1391.06 ± 9.28 ^c^
Leucine	4219.74 ± 0.93 ^a^	4070.86 ± 29.68 ^b^	2421.40 ± 17.62 ^c^
Lysine	3651.46 ± 5.69 ^a^	3590.79 ± 30.49 ^a^	1973.57 ± 17.57 ^c^
Methionine	947.86 ± 0.53 ^a^	832.96 ± 4.74 ^b^	587.63 ± 8.49 ^c^
Phenylalanine	2015.32 ± 4.67 ^a^	1936.03 ± 24.53 ^a^	1239.75 ± 42.05 ^b^
Proline	3925.65 ± 24.02 ^a^	3751.22 ± 27.27 ^a^	1999.52 ± 109.50 ^c^
Serine	2567.61 ± 4.83 ^a^	2500.62 ± 20.81 ^b^	1440.52 ± 16.72 ^c^
Threonine	2342.49 ± 10.11 ^a^	2298.12 ± 18.95 ^a^	1284.71 ± 31.17 ^b^
Tyrosine	2769.48 ± 17.93 ^a^	2610.07 ± 26.88 ^b^	1658.07 ± 7.94 ^c^
Valine	2692.69 ± 19.05	2558.26 ± 16.16	ND

Note: Means in the same row with different letters are considered statistically significantly different at the 95 percent confidence level (*p* < 0.05). ND: not detected.

## Data Availability

The original contributions presented in the study are included in the article/supplementary material, further inquiries can be directed to the corresponding author.

## Supplementary Materials

The following supporting information can be downloaded at: https://www.mdpi.com/article/10.3390/foods13162486/s1, Figure S1: Quercetin standard curve in methanol (15.625–500 μg/mL); Figure S2: Chromatogram of quercetin at a retention time of 8.232 min (Rt = 8.232); Figure S3: Chromatogram of an extract from the tray-dried bee brood sample with quercetin at retention time 8.237 min (Rt = 8.237); Figure S4: Chromatogram of bee brood oil extracted via supercritical CO_2_ (extraction at 50 °C, 600 bar for 1 h) with quercetin at retention time 8.230 min (Rt = 8.230).
