# Supplementary material for: Utilizing Supercritical CO2 for Bee Brood Oil Extraction and Analysis of Its Chemical Properties
Source: Foods. 2024 Aug 8;13(16):2486. doi: 10.3390/foods13162486 (PMC11354136; doi:10.3390/foods13162486)
Supplement: Supplementary file 1 [file foods-13-02486-s001.zip › foods-3125444-supplementary.pdf]

Supplementary Figures

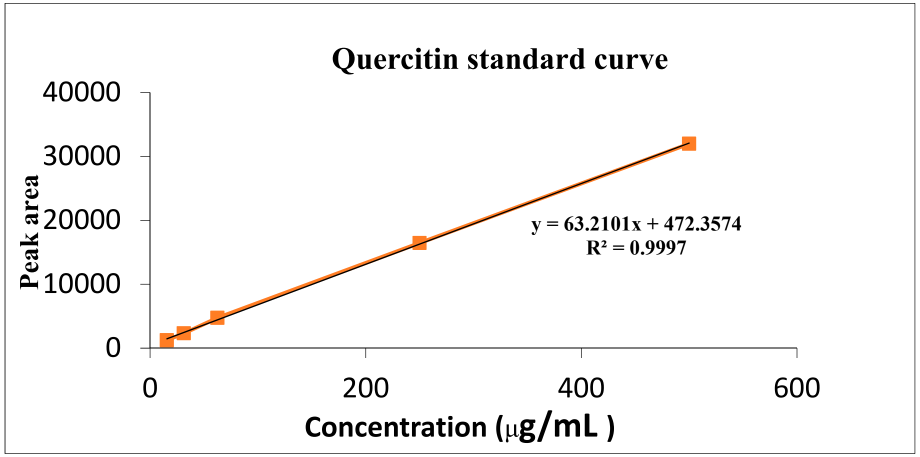

Supplemental Figure S1. Quercetin standard curve in methanol (15.625–500 μg/mL).

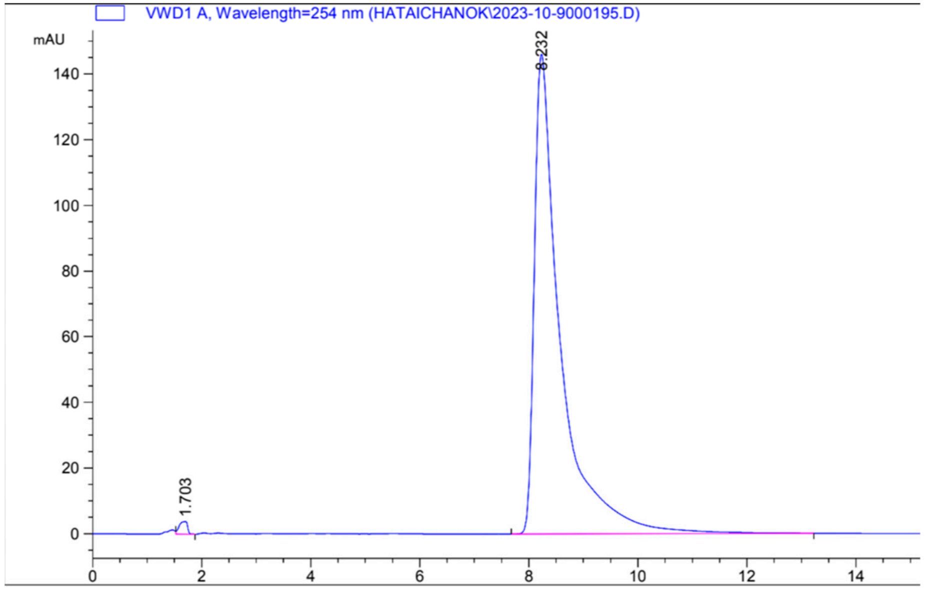

Supplemental Figure S2. Chromatogram of quercetin at retention time 8.232 minutes (Rt = 8.232).

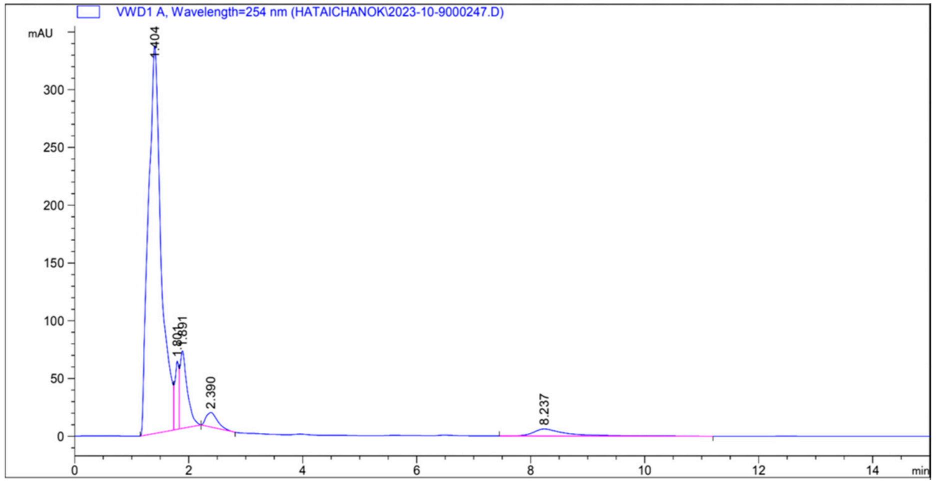

Supplemental Figure S3. Chromatogram of extract from tray-dried bee brood sample with quercetin at retention time 8.237 minutes (Rt = 8.237).

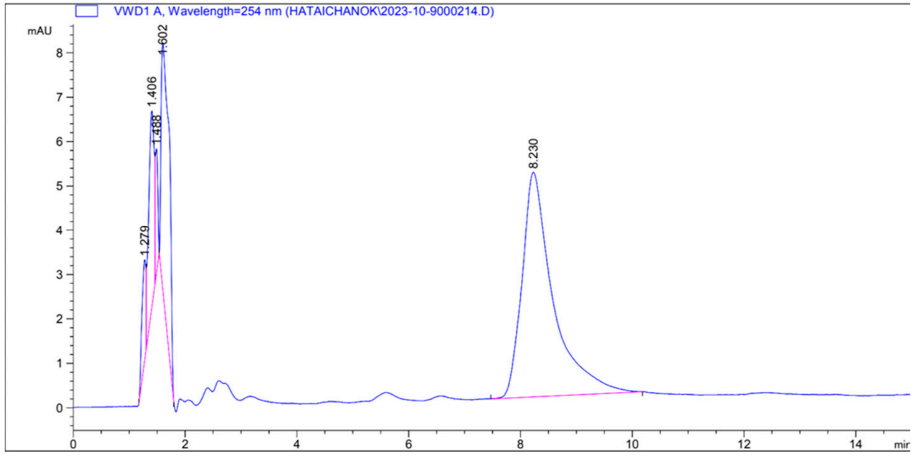

Supplemental Figure S4. Chromatogram of bee brood oil extracted via supercritical CO_2 (extraction at 50 ºC, 600 bar for 1 hour) with quercetin at retention time 8.230 minutes (Rt = 8.230).
